# Combination of *G72* Genetic Variation and G72 Protein Level to Detect Schizophrenia: Machine Learning Approaches

**DOI:** 10.3389/fpsyt.2018.00566

**Published:** 2018-11-06

**Authors:** Eugene Lin, Chieh-Hsin Lin, Yi-Lun Lai, Chiung-Hsien Huang, Yu-Jhen Huang, Hsien-Yuan Lane

**Affiliations:** ^1^Department of Electrical & Computer Engineering, University of Washington, Seattle, WA, United States; ^2^Department of Biostatistics, University of Washington, Seattle, WA, United States; ^3^Graduate Institute of Biomedical Sciences, China Medical University, Taichung, Taiwan; ^4^Department of Psychiatry, Kaohsiung Chang Gung Memorial Hospital, Chang Gung University College of Medicine, Kaohsiung, Taiwan; ^5^School of Medicine, Chang Gung University, Taoyuan, Taiwan; ^6^Department of Medicine Research, China Medical University Hospital, Taichung, Taiwan; ^7^Department of Psychiatry, China Medical University Hospital, Taichung, Taiwan; ^8^Brain Disease Research Center, China Medical University Hospital, Taichung, Taiwan; ^9^Department of Psychology, College of Medical and Health Sciences, Asia University, Taichung, Taiwan

**Keywords:** artificial intelligence, D-amino acid oxidase activator, G72, machine learning algorithm, schizophrenia, single nucleotide polymorphism

## Abstract

The *D-amino acid oxidase activator* (*DAOA*, also known as *G72*) gene is a strong schizophrenia susceptibility gene. Higher G72 protein levels have been implicated in patients with schizophrenia. The current study aimed to differentiate patients with schizophrenia from healthy individuals using *G72* single nucleotide polymorphisms (SNPs) and G72 protein levels by leveraging computational artificial intelligence and machine learning tools. A total of 149 subjects with 89 patients with schizophrenia and 60 healthy controls were recruited. Two *G72* genotypes (including rs1421292 and rs2391191) and G72 protein levels were measured with the peripheral blood. We utilized three machine learning algorithms (including logistic regression, naive Bayes, and C4.5 decision tree) to build the optimal predictive model for distinguishing schizophrenia patients from healthy controls. The naive Bayes model using two factors, including *G72* rs1421292 and G72 protein, appeared to be the best model for disease susceptibility (sensitivity = 0.7969, specificity = 0.9372, area under the receiver operating characteristic curve (AUC) = 0.9356). However, a model integrating *G72* rs1421292 only slightly increased the discriminative power than a model with G72 protein alone (sensitivity = 0.7941, specificity = 0.9503, AUC = 0.9324). Among the three models with G72 protein alone, the naive Bayes with G72 protein alone had the best specificity (0.9503), while logistic regression with G72 protein alone was the most sensitive (0.8765). The findings remained similar after adjusting for age and gender. This study suggests that G72 protein alone, without incorporating the two *G72* SNPs, may have been suitable enough to identify schizophrenia patients. We also recommend applying both naive Bayes and logistic regression models for the best specificity and sensitivity, respectively. Larger-scale studies are warranted to confirm the findings.

## Introduction

Schizophrenia is a severe mental disorder characterized by symptoms such as delusions, hallucinations, blunted affect, impaired executive function, reduced motivation, and disorganized communication (1). The prevalence of schizophrenia is around 1% worldwide, and the social and economic costs of schizophrenia are enormous (2, 3). Converging evidence from genome-wide linkage studies, genetic case-control association studies, and genome-wide association studies indicate that several potential candidate genes are associated with schizophrenia (4). More and more genetic studies have employed novel computational tools such as naive Bayes to conduct gene discovery and detect new gene loci associated with schizophrenia (5). Identification of susceptibility genes for schizophrenia will help in early detection and prevention of high-risk individuals, as well as in developing novel therapies (6). The *D-amino-acid oxidase activator* (*DAOA*, also named *G72*) gene is one of the candidate genes.

The *G72* gene, located on chromosome 13q3, exists in exclusively four primate species (7). Furthermore, the *G72* gene encodes the protein that has been shown to function as a putative activator of D-amino acid oxidase (DAO), located in peroxisomes (7) and a mitochondrial protein (8). *In vitro* studies also demonstrate that the G72 protein binds to and activates DAO, which is capable of oxidizing D-amino acids such as D-serine, an agonist of the N-methyl-D-aspartate receptor (NMDAR) (7, 9). The agonist activity at NMDAR may have particular relevance to a novel drug target for treatment of schizophrenia (10–16). One hypothesis of schizophrenia is that individuals who overproduce the G72 protein have lower D-amino acid levels and reduced NMDAR activity, predisposing them to schizophrenia (17, 18). A study suggests that the plasma G72 protein levels may be distinctively higher in patients with schizophrenia than healthy individuals (18). Of note, G72 protein levels are very similar between the medicated patients and the drug-free patients, implying that antipsychotic treatment does not influence G72 levels in plasma (18). In addition, *G72* transgenic mice studies indicate a role of *G72* in modulating behaviors relevant to schizophrenia (19–21). The *G72* gene was also reported to predispose to schizophrenia in French Canadian (7), Russian (7), Chinese (22–24), German (25), and Ashkenazi (26) populations in single nucleotide polymorphism (SNP)-based studies.

A pilot study (18) modeled disease susceptibility to schizophrenia with plasma G72 protein levels using logistic regression. The current larger-sized study compared three artificial intelligence and machine learning techniques (including logistic regression, naive Bayes, and C4.5 decision tree) in predicting schizophrenia using G72 protein levels plus *G72* SNPs. These three artificial intelligence and machine learning algorithms were chosen because they are well-known techniques with distinctively representational models; regression models for logistic regression (27), probabilistic models for naive Bayes (28), and decision tree models for the C4.5 algorithm (29).

## Materials and methods

### Study population

This study was approved by the institutional review board of China Medical University Hospital, Taiwan, and carried out in accordance with the Declaration of Helsinki. Consecutive patients were screened and recruited from the psychiatric treatment programs of China Medical University Hospital, which is a major medical center in Taiwan. The patient population is similar to that of other mental health facilities. After complete description of the study to the subjects, written informed consents were obtained in line with the institutional review board guidelines. The study subjects were partially original to a previous study (18); the same 60 healthy individuals, but with more schizophrenia patients.

In the cohort, both patients and controls were Han Chinese aged 18–50 years, who were physically and neurologically healthy and had normal laboratory assessments (including urine/blood routine and biochemical tests). Both patients and controls were evaluated by the research psychiatrists using the Structured Clinical Interview for DSM-IV (SCID) for diagnosis. All patients had a DSM-IV diagnosis of schizophrenia. Patients with Axis I diagnosis other than schizophrenia, or any Axis II diagnosis were not included. All healthy volunteers were free of any Axis I or II psychiatric disorder. To exclude potential confounding effects, all participants were non-smokers and had no DSM-IV diagnosis of substance (including alcohol) abuse or dependence.

Drug history was ascertained by interviewing the patients and family members or caregivers, contacting other health care providers, and reviewing chart. Healthy controls had no history of exposure to psychotropic agents. Among schizophrenia patients, some patients were psychotropic-free for 3 months or longer and the other patients were stabilized on antipsychotics (risperidone, zotepine, haloperidol, quetiapine, amisulpride, sulpiride, flupentixol, olanzapine, ziprasidone, chlorpromazine, or paliperidone) for at least 3 months (18). The G72 protein level was not correlated with the medications administrated by patients (18).

### Laboratory assessments: genotyping

DNA was isolated from blood samples using MasterPure DNA purification kit following the manufacturer's instructions (EPICENTRE, Madison, Wisconsin, USA). To extract DNA, we used 200 μl of blood which was further solved in 100 μl of distilled water (30). The extracted DNA was diluted to the concentration of 50 ng/μl determined by the absorbance at 260 nM (ND-1000 UV-Vis spectrophotometer, Thermo Fisher Scientific Inc.). Four standard DNA samples with known genotypes were used for quality control (31).

All SNP genotyping was performed using the Taqman SNP genotyping assay (ABI: Applied Biosystems Inc., Foster City, CA, USA). The primers and probes of SNPs were provided by the ABI Company. The PCR reaction was conducted in 15 μl reaction volume which contained 0.4 μl DNA sample (50 ng), 7.5 μl Master mix (Roche), and 0.4 μl 40x primer pairs and probes. The samples were pre-incubated at 95°C for 10 min to activate the Hot-Start DNA polymerase and to denature DNA, following by 40 amplification cycles of 92°C denaturation for 15 s and 60°C for 60 s. The probe fluorescence signal detection was performed using the ABI Prism 7500 Real-Time PCR System.

### Laboratory assessments: western blotting

The plasma G72 protein expression levels were examined by western blotting (18). Ten milliliter of blood was collected into EDTA-containing blood collection tubes by personnel trained in phlebotomy using sterile technique. The blood specimens were processed immediately by centrifugation at 500 g. After centrifugation, plasma was quickly dissected and immediately stored at −80°C until western blotting.

For western blotting, 100 μl plasma was depleted using ProteoPrep® Blue Albumin and IgG Depletion Kit (Sigma). The low-abundant protein fractions were collected to 100 μl. Then, 10 μl of the fractions were mixed with 4X sample buffer (500 mM Tris-HCl (pH 6.8), 16% SDS, 80% glycerol, 400 mM DTT, and 0.08% bromophenol blue) and separated on 12% SDS-PAGE. Proteins in the gels were transferred to 0.45 μm polyvinylidene difluoride (PVDF) membrane (Millipore). The membranes were placed in 5% nonfat dry milk in TBST (20 mM Tris-HCl pH 7.6, 500 mM sodium chloride, 0.1% Tween 20) for 1 h at room temperature, then incubated with goat anti-G72 antibody (G72(N15):sc-46118, Santa Cruz Biotechnology) diluted by 1:1,000 in TBST overnight at 4°C. The membranes were washed for 3 times in TBST and incubated for 2 h with a HRP-linked anti-goat IgG secondary antibody (sc-2030, Santa Cruz Biotechnology) diluted by 1:5,000 in TBST. After 3 washes in TBST, the blots were visualized with an ECL Advance Western Blotting Detection Kit (RPN2135, GE Healthcare). The stained membranes were photographed on ImageQuant LAS 4000 mini (GE Healthcare) and quantified using ImageQuant™ TL 7.0 software (GE Healthcare) by measuring the relative intensity from each band and normalized to the G72 recombinant protein (20 ng) signals. All western blot analyses were repeated for two times.

### Machine learning algorithms

Machine learning algorithm is a procedure for choosing the best hypothesis from a set of alternatives that fit a set of observations (27, 32). The advantages of machine learning algorithms, including nonlinearity, fault tolerance, and real-time operation, make them suitable for complex applications (33). The current study employed three families of machine learning algorithms, including logistic regression, naive Bayes, and C4.5 decision tree. Logistic regression analysis, the standard method for clinical classification (27), was used as a basis for comparison. The analyses were performed using the Waikato Environment for Knowledge Analysis (WEKA) software (27).

The naive Bayes classifier assumes that the presence or absence of a particular feature is unrelated to the presence or absence of any other feature (27). It calculates the probability that a given instance belongs to a certain class (“schizophrenia“ or “control“ in this study) by using Bayes' theorem.

The C4.5 decision tree is a model which builds decision trees top-down and prunes them using the concept of information entropy (27). The tree is first constructed by finding the root node (SNP or protein level) that is most discriminative for differentiating a disease status from “control.” The best single feature test is decided by the information gain from choosing a feature (SNP or protein level) to split the data into subsets. Here, we used the default parameters of WEKA, such as 0.25 for the confidence factor and 2 for the minimum number of instances per leaf node (34).

### Evaluation of the predictive performance

The repeated 10-fold cross-validation method was used to investigate the generalization of the predictive models produced by the aforementioned algorithms (34–36). To measure the performance of the predictive models, we used the receiver operating characteristic (ROC) method and calculated the area under curve (AUC) to compare the performances of different predictive models (34, 35). AUC is a better performance metric than accuracy; the higher AUC means the better performance (36).

### Statistical analysis

We analyzed the categorical data using the chi-square test. Differences for continuous variables were compared using the Student's *t*-test (37). Genotype frequencies were evaluated for Hardy-Weinberg equilibrium using a χ^2^ goodness-of-fit test. The criterion for significance was set at *P* < 0.05 for all tests. Data are presented as mean ± standard deviation (SD).

## Results

### Findings from the unmatched sample

The participants were 60 unrelated healthy individuals and 89 schizophrenia patients. As shown in Table 1, there was no significant difference in gender distribution between the two groups. The mean age (37.8 ± 10.5) of schizophrenia patients was older than that of healthy controls (32.8 ± 9.9, *P* = 0.004). The mean level of G72 protein in the plasma of schizophrenia patients was markedly higher than that of healthy controls (4.057 ± 2.594 ng/μL vs. 1.147 ± 0.574 ng/μL, respectively, *P* < 0.0001) (Table 1). The genotype frequencies for both rs1421292 and rs2391191 of the *G72* gene were in Hardy–Weinberg equilibrium (*P* = 0.39 and 0.27, respectively).

**Table 1:** Demographic characteristics of schizophrenia patients and unmatched healthy individuals.

**Parameter**	**Healthy individuals**	**Schizophrenia patients**	***P*-value ^a^**
N	60	89	
Gender			0.825
Male	36 (61.9%)	55 (70.4%)	
Female	24 (38.1%)	34 (29.6%)	
Age (year), mean (SD)	32.8 ± 9.9	37.8 ± 10.5	0.004
Education (year)	15.1 ± 2.2	11.5 ± 2.0	<0.0001
Age at onset (year)		22.9 ± 6.1	
Illness duration (m)		169.3 ± 109.3	
PANSS total score		94.8 ± 18.6	
G72 level (ng/μL)	1.147 ± 0.574	4.057 ± 2.594	<0.0001

*PANSS: Positive and Negative Syndrome Scale*.

a *Chi-square test for the categorical data; Student's t-test for continuous variables*.

#### AUC after adding rs1421292 was only slightly better than that from G72 protein alone

Table 2 summarizes the results from the naive Bayes algorithm. We generated five models (Models 1–5) with various combinations of three factors (rs2391191, rs1421292, and G72 protein levels). Among the five models, Model 2 with rs1421292 and G72 protein levels had the best AUC. Its AUC, sensitivity, and specificity were 0.9356, 0.7969, and 0.9372, respectively (Table 2). However, the AUC value after adding rs1421292 was only slightly better than that of G72 protein alone by 0.32% (Model 2 vs. Model 5).

**Table 2:** Five naive Bayes models for differentiating schizophrenia patients from unmatched healthy individuals.

**Model**	**AUC**	**Sensitivity**	**Specificity**	**Number of factors**
(1) Using G72 protein, rs1421292, rs2391191	0.9280	0.7945	0.9213	3
(2) Using G72 protein, rs1421292	0.9356	0.7969	0.9372	2
(3) Using G72 protein, rs2391191	0.9244	0.7924	0.9320	2
(4) Using rs1421292, rs2391191	0.4612	0.9704	0.0070	2
(5) Using G72 protein	0.9324	0.7941	0.9503	1

We then employed the C4.5 decision tree algorithm with the same three factors (Table 3). Among the five models, Model 7 with rs1421292 and G72 protein levels had the best AUC. Its AUC, sensitivity, and specificity were 0.8525, 0.8202, and 0.8843, respectively (Table 3). The AUC value after adding rs1421292 was only slightly better than that of G72 protein alone by 0.19% (Model 7 vs. Model 10).

**Table 3:** Five C4.5 decision tree models for differentiating schizophrenia patients from unmatched healthy individuals.

**Model**	**AUC**	**Sensitivity**	**Specificity**	**Number of factors**
(6) Using G72 protein, rs1421292, rs2391191	0.8515	0.8236	0.8772	3
(7) Using G72 protein, rs1421292	0.8525	0.8202	0.8843	2
(8) Using G72 protein, rs2391191	0.8504	0.8275	0.8725	2
(9) Using rs1421292, rs2391191	0.5000	1.0000	0.0000	2
(10) Using G72 protein	0.8506	0.8274	0.8725	1

We finally tested the same factors with logistic regression (Table 4). The AUC, sensitivity, and specificity for the best logistic regression model (Model 12, applying rs1421292, and G72 protein levels) were 0.9272, 0.8576, and 0.8923, respectively. The AUC value only slightly increased by 0.97% after adding rs1421292 (Model 12 vs. Model 15).

**Table 4:** Five logistic regression models for differentiating schizophrenia patients from unmatched healthy individuals.

**Model**	**AUC**	**Sensitivity**	**Specificity**	**Number of factors**
(11) Using G72 protein, rs1421292, rs2391191	0.9200	0.8567	0.8400	3
(12) Using G72 protein, rs1421292	0.9272	0.8576	0.8923	2
(13) Using G72 protein, rs2391191	0.9107	0.8713	0.8607	2
(14) Using rs1421292, rs2391191	0.4533	0.9619	0.0088	2
(15) Using G72 protein	0.9175	0.8765	0.8577	1

#### Of the G72 protein models, naive bayes was specific; and logistic regression, sensitive

Among all the 15 models (Models 1–15) with unmatched schizophrenia patients and healthy controls, the naive Bayes (Model 2) with rs1421292 and G72 protein levels had the highest AUC. Of the three models with G72 protein alone (Models 5, 10, and 15), the naive Bayes (Model 5) had the best specificity (0.9503) and logistic regression (Model 15) had the best sensitivity (0.8765).

We further tested the relationship between *G72* genotypes and G72 protein levels. The distribution of the two SNPs [for example, the numbers of TT (*n* = 57), TA (*n* = 65), and AA (*n* = 27) carriers in rs1421292] was illustrated in Table 5. As shown in Table 5, the G72 protein levels were marginally higher in the subjects with the TT or TA genotype than the AA homozygotes for rs1421292 (3.051 ± 2.588 vs. 2.137 ± 1.819; *P* = 0.084). There was no association between genetic variances of rs2391191 and G72 protein levels.

**Table 5:** Relationship between *G72* genotypes and G72 protein level with schizophrenia patients and unmatched healthy individuals.

***G72* rs1421292**	**AA**	**TT**	**TA**	**TT + TA**	***P*-value^a^**	***P*-value^b^**	***P*-value^c^**
N	27	57	65	122			
G72 protein level, mean (SD)	2.137 ± 1.819	3.219 ± 3.032	2.903 ± 2.139	3.051 ± 2.588	0.09	0.11	0.084
***G72*** **rs2391191**	**AA**	**GG**	**AG**	**GG** + **AG**	***P*****-value**^d^	***P*****-value**^e^	***P*****-value**^f^
N	63	22	64	86			
G72 protein level, mean (SD)	2.586 ± 2.083	3.570 ± 3.356	2.943 ± 2.499	3.104 ± 2.736	0.11	0.38	0.21

a *P value for comparing the subjects of the AA genotype with those of the TT genotype*.

b *P value for comparing the subjects of the AA genotype with those of the TA genotype*.

c *P value for comparing the subjects of the AA genotype with those of the TT or TA genotype*.

d *P value for comparing the subjects of the AA genotype with those of the GG genotype*.

e *P value for comparing the subjects of the AA genotype with those of the AG genotype*.

f *P value for comparing the subjects of the AA genotype with those of the GG or AG genotype*.

### Findings from the matched sample

Next, we selected 66 patients from the schizophrenia group to match better with healthy controls by age. The demographic characteristics of age and gender-matched schizophrenia patients and healthy controls are shown in Table 6. There was no significant difference in gender and age distributions between the two groups. The G72 levels in the plasma of schizophrenia patients were markedly higher than that of the matched healthy controls (4.188 ± 2.772 ng/μL and 1.147 ± 0.574 ng/μL, respectively, *P* < 0.0001) (Table 6).

**Table 6:** Demographic characteristics of schizophrenia patients and matched healthy individuals.

**Parameter**	**Healthy individuals**	**Schizophrenia patients**	***P*-value ^a^**
N	60	66	
Gender			0.783
Male	36 (61.9%)	38 (57.6%)	
Female	24 (38.1%)	28 (42.4%)	
Age (year), mean (SD)	32.8 ± 9.9	33.2 ± 7.2	0.820
Education (year)	15.1 ± 2.2	11.6 ± 2.0	<0.0001
Age at onset (year)		21.3 ± 5.5	
Illness duration (m)		141.1 ± 95.7	
PANSS total score		95.9 ± 19.3	
G72 level (ng/μL)	1.147 ± 0.574	4.188 ± 2.772	<0.0001

*PANSS: Positive and Negative Syndrome Scale*.

a *Chi-square test for the categorical data; Student's t-test for continuous variables*.

#### The findings from the matched sample were similar to those from the unmatched sample

Table 7 shows the analytic results of schizophrenia patients and matched healthy controls. The findings from the matched sample were similar to those from the unmatched sample. Among the three models (Models 16–18) with G72 protein alone, the naive Bayes model (Model 16) performed best in specificity (0.966), and logistic regression (Model 18) had the best sensitivity (0.8483) (Table 7).

**Table 7:** The models of naive Bayes, C4.5 decision tree, and Logistic regression for differentiating schizophrenia patients from matched healthy individuals using G72 protein.

**Model**	**AUC**	**Sensitivity**	**Specificity**	**Number of factors**
(16) Naive Bayes with G72 protein	0.9396	0.7914	0.9660	1
(17) C4.5 decision tree with G72 protein	0.8510	0.7871	0.9152	1
(18) Logistic regression with G72 protein	0.9099	0.8483	0.9072	1

## Discussion

To our knowledge, this is the first study to examine the relationships between schizophrenia and *G72* SNPs plus plasma G72 protein levels. We compared three machine learning algorithms, including logistic regression, naive Bayes, and C4.5 decision tree, in differentiating schizophrenia patients from healthy individuals. The results showed that the naive Bayes with *G72* rs1421292 SNP and G72 protein levels (Model 2) performed best among all models (Models 1–15). The combination of *G72* rs1421292 SNP and G72 protein levels was also the best model using the C4.5 decision tree (Model 7) and logistic regression (Model 12). These results were consistent with another finding of this study; that is, *G72* rs1421292 SNP was marginally associated with G72 protein levels (Table 5). The proposed procedures can be implemented using the publicly available software WEKA (27) and thus can be widely used in genomic studies.

However, the AUC value after adding rs1421292 was only slightly better than that of G72 protein alone by an increase of 0.32% (Model 2 vs. Model 5) and 0.97% (Model 12 vs. Model 15), respectively. Hence, the present study suggests that G72 protein alone may have been feasible enough in AUC. Moreover, among the three models with G72 protein alone, logistic regression performed best in sensitivity, and the naive Bayes model was the most specific. This finding remained similar in the matched sample. We therefore recommend a combination model using logistic regression (for sensitivity) and naive Bayes (for specificity).

The common SNPs, such as rs2391191 and rs1421292, of the *G72* gene have received considerable attention. These two SNPs were shown to be associated with schizophrenia (7, 23–25); however, the findings are discordant. The rs2391191 SNP was reported to predispose to schizophrenia in Chinese (23, 24) and German (25) subjects, but not in French Canadian (7), United States (38), Scottish (22), Chinese (22, 39), and Taiwanese (40) populations. On the other hand, the rs1421292 SNP was found to be associated with schizophrenia in French Canadian (7), Russian (7), and German (25) subjects, but not in Japanese sample (41), UK population (42) and a mix of different races (including 84% Caucasian and 9% African American) in the United States (38). Moreover, two recent genome-wide association studies (GWAS) have been conducted to identify susceptible genetic loci affecting schizophrenia in mainly European (43) and Chinese (44) populations, respectively. However, no association of schizophrenia with SNPs in the *G72* gene was found in these two large GWAS. Furthermore, by utilizing expression quantitative trait loci (eQTL) analyses, one of these GWAS implicated that there was no genetic risk regulating gene expression of *G72* effect in brain or blood when eQTL analyses were used to explain associations with schizophrenia (43). The current study showed that the models which combined rs2391191 and rs1421292 (Models 4, 9, and 14) were not as good as other models (Tables 2–4). Moreover, adding rs2391191 or rs1421292 could not increase the AUC significantly than G72 protein alone. In agreement with several previous studies (7, 22, 38–44), the current study with a small sample size didn't demonstrate an association between the two *G72* SNPs and schizophrenia.

In our previous study (18) on G72 protein levels, the severity of disease, the medications administrated by patients, as well as illness duration of the medicated patients did not influence the G72 protein level. In addition, the G72 protein level was significantly associated with schizophrenia in multivariate logistic regression analyses (18). The G72 protein level was also higher in drug-free or medicated schizophrenia patients than in healthy controls (18). The current larger-sized study extended the previous study by combining G72 protein levels with *G72* SNPs as well as by leveraging the state-of-the-art artificial intelligence and machine learning algorithms. The current results implicated that the relevance of G72 protein levels to schizophrenia is much more significant than that of SNPs.

This study has several limitations. First, we chose only two SNPs of *G72* for the current study because they seem to be two most commonly used SNPs. Whether other SNPs of *G72* (25) could contribute more in the models of predicting schizophrenia remains unknown. Second, the findings of the current study came from a single population. More studies are necessary to testify whether the findings could be replicated in non-Taiwanese subjects (45, 46). Third, the small sample size does not allow us to draw definite conclusions (46). In the future, large-scale prospective studies in other ethnicities are warranted to reconfirm the potential of G72 protein level and *G72* SNPs as the biomarkers for schizophrenia.

## Conclusions

In conclusion, this preliminary study tested and compared numerous models using machine learning algorithms for predicting schizophrenia. The findings suggest that the models with G72 protein alone, without adding *G72* SNPs, may have good enough power to discriminate patients with schizophrenia from healthy individuals. We also propose a combination of logistic regression and naive Bayes models to build a both sensitive and specific model to predict schizophrenia. Independent replications with larger-scale studies in other racial populations are needed to confirm the role of the *G72* SNPs and G72 protein found in the current study.

## Author contributions

EL, C-HL, and H-YL designed the study. EL analyzed the data. EL, C-HL, and H-YL drafted and revised the manuscript. Y-LL, Y-JH, and C-HH conducted the laboratory experiments. All authors provided the final approval of the version to be published.

### Conflict of interest statement

The authors declare that the research was conducted in the absence of any commercial or financial relationships that could be construed as a potential conflict of interest.
